# Relationship between acid–base status and inflammation in the critically ill

**DOI:** 10.1186/cc13993

**Published:** 2014-07-17

**Authors:** Fernando G Zampieri, John A Kellum, Marcelo Park, Otavio T Ranzani, Hermes V Barbeiro, Heraldo P de Souza, Luiz Monteiro da Cruz Neto, Fabiano Pinheiro da Silva

**Affiliations:** 1 Intensive Care Unit, Emergency Medicine Discipline, Hospital das Clínicas, University of São Paulo, São Paulo, Brazil; 2 Laboratory of Medical Investigation of the Emergency Medicine Discipline (LIM-51), University of São Paulo, São Paulo, Brazil; 3 Center for Critical Care Nephrology, University of Pittsburgh, Pittsburgh, Pennsylvania, USA; 4 CRISMA (Clinical Research, Investigation, and Systems Modeling of Acute Illness) Center, Department of Critical Care Medicine, University of Pittsburgh, Pittsburgh, Pennsylvania, USA; 5 Intensive Care Unit, Hospital Alemão Oswaldo Cruz, São Paulo, Brazil

## Abstract

**Introduction:**

There is a complex interplay between changes in acid–base components and inflammation. This manuscript aims to explore associations between plasma cytokines and chemokines and acid–base status on admission to intensive care.

**Methods:**

We conducted a prospective cohort study in a 13-bed ICU in a tertiary-care center in Brazil. 87 unselected patients admitted to the ICU during a 2-year period were included. We measured multiple inflammatory mediators in plasma using multiplex assays and evaluated the association between mediator concentrations and acid–base variables using a variety of statistical modeling approaches, including generalized linear models, multiadaptive regression splines and principal component analysis.

**Results:**

We found a positive association between strong ion gap (SIG) and plasma concentrations of interleukin (IL)6, 8, 10 and tumor necrosis factor (TNF); whereas albumin was negatively associated with IL6, IL7, IL8, IL10, TNF and interferon (IFN)α. Apparent strong ion difference (SID_a_) was negatively associated with IL10 and IL17. A principal component analysis including SAPS 3 indicated that the association between acid–base components and inflammatory status was largely independent of illness severity, with both increased SIG and decreased SID_a_ (both drivers of acidosis) associated with increased inflammation.

**Conclusion:**

Acid–base variables (especially increased SIG, decreased albumin and decreased SID_a_) on admission to ICU are associated with immunological activation. These findings should encourage new research into the effects of acid–base status on inflammation.

## Introduction

Organ dysfunction during sepsis and related conditions is generally understood to arise as a consequence both of the triggering event (for example, infection) and the host response [1,2]. One of the key aspects of the host response is the production and systemic release of cytokines and other inflammatory mediators [2]. Cytokine release is induced by a variety of signals, including those derived from pathogens (for example, endotoxin), or those related to tissue damage (for example, complement activation, ischemia-reperfusion injury and oxidative stress) [3]. These mediators, in turn, drive pro-/anti-inflammatory responses and can ultimately alter the clinical trajectory toward the resolution of the illness or result in ongoing organ damage and death [4].

A complex interplay between acid-base abnormalities and inflammation has been suggested [5-7]. Acid-base abnormalities are common in critically ill patients and may be evaluated at the bedside using the physicochemical approach [8,9], the components of which, including the strong ion gap (SIG) may have both prognostic [10,11] and treatment implications [12]. SIG is an accurate estimator of unmeasured anions [13]. Although evidence from cell culture [14] and intact animals [15] show that acidosis increases inflammation, it is unknown if a specific change in acid-base status (for example, increase in SIG or decrease in weak acids such as albumin) in humans is associated with changes in inflammatory response. It has been suggested that albumin could alter the inflammatory response by a number of ways, including scavenging and modulation of cytokine production [16,17]. No study hitherto has evaluated the association between acid-base abnormalities (for example high SIG, low albumin) in critically ill humans and inflammatory mediator expression.

This study aimed to evaluate the association between various acid-base parameters [18,19] and plasma inflammatory mediator expression on admission to the ICU. We hypothesized that there would be an association between acid-base status and plasma cytokines, with a greater degree of inflammation being associated with higher SIG and lower albumin. We also sought to evaluate the association between cytokine values and organ dysfunction (specifically acute kidney injury (AKI) and shock) in the first 48 hours after ICU admission.

## Methods

### Patients and study design

After obtaining approval from Hospital das Clínicas ethics committee (approval number 1207/09), we conducted a prospective cohort study in which we enrolled 87 critically ill patients admitted to an ICU in a tertiary-care center in a large university hospital in São Paulo, Brazil, from February 2010 to January 2012. After informed consent was obtained from the patient or legal representative, blood was collected on the morning after ICU admission. General laboratory data were collected at the same time, as was blood for cytokine measurement, and these samples were then processed by the clinical laboratory.

### Fluid expansion policy

Fluid expansion prior to ICU admission was performed at the discretion of the attending physician. Lactated Ringer’s is the fluid of choice for expansion in our emergency department and in the ICU; saline is recommended only for patients with traumatic brain injury. Due to its costs, albumin is only used in specific clinical conditions, such as burns, cirrhosis and hepatorenal syndrome. Other colloids, including starches, are not available for use at our institution.

### Cytokine measurement

We measured a panel of 15 inflammatory mediators (cytokines and chemokines) using Miliplex® technology (Merck, Genese diagnostics, Darmstadt, Germany), a multiplex method for cytokine analysis. Our panel included multiple interleukins (IL) including IL1β, IL2, IL4, IL6, IL7, IL8, IL9, IL10, IL17, TNF, IFNα, vascular endothelial growth factor (VEGF), chemokine CXC motif ligand (CXCL)1, and monocyte chemoattractant protein (MCP)1.

### Calculation of acid-base variables

In order to calculate strong ion gap (SIG), both the apparent and effective strong ion difference (SID_a_ and SID_e_) were calculated as previously described [11,13]. SID_a_ was calculated as:

$$\left(Na^{+}\right)\;+\;\left(K^{+}\right)\;+\;\left(Mg^{2+}\right)\;+\;\left(Ca^{2+}\right)\;–\;\left(Cl^{‒}\right)\;–\;\left(\mathrm{Lactat}e^{‒}\right)$$

SID_e_ was calculated as:

$$\begin{array}{c} 2.46\;\times \;10^{‒8}\times \;\mathrm{pCO}2/\left(10^{‒\mathrm{pH}}\right)\;\times \;\left(\mathrm{Albumin}\right)\;\\ \;\times \;\left(0.123\;\times \;\mathrm{pH}\;–\;0.631\right)\;+\;\left(P{O_{4}}^{‒}\right)\;\\ \;\times \;\left(0.309\;\times \;\mathrm{pH}\;–\;0.469\right) \end{array}$$

Finally, SIG was calculated as:

$$\mathrm{SI}D_{a}–\;\mathrm{SI}D_{e}$$

Blood gas analysis and lactate measurement were performed using the OMNI analyzer (Roche Diagnostics System, F Hoffmann, La Roche Ltd, Basel, Switzerland).

### Statistical analysis

Data were tested for normality using the Kolmogorov-Smirnoff or Shapiro-Wilkes test as appropriate. Continuous normal data were compared using the *t*-test or analysis of variance. Continuous non-parametric data were compared with Mann-Whitney or Kruskal-Wallis test, as appropriate. Fisher’s exact test or the Chi-squared test was used for dichotomous variables.

To evaluate the association between mediator concentrations and components of acid-base status we fit a generalized linear model (GLM) using each measured mediator as the dependent variable and components of acid-base status (partial pressure of CO_2_ – PCO_2_, SIG, SID_a_, albumin, lactate and phosphate concentrations) as variables. In order to use a Gaussian distribution for the GLM, mediator concentrations were log-transformed prior to analysis and we used log as a link function in the GLM. All variables were included in the model.

We also performed multivariate adaptive regression with splines (MARS) to evaluate the association of selected cytokines and acid-base components. We limited this analysis to mediators previously shown to be strongly associated with outcomes in patients with sepsis (IL6, IL10) [6] and in those associated with organ dysfunction [20] (MCP1, IL8: see online data supplement). MARS has the advantage of automatically modeling non-linearity and interactions between variables. A MARS model is built on forward and backwards steps; generalized cross-validation is used for the backward analysis. The results of MARS analysis include an intercept value, hinge function and products of two or more hinge functions to account for the interplay between variables. Therefore, MARS models are useful for handling databases when association is not linear and when multiple interactions between variables are expected [21].

Illness severity is associated with inflammation and may interfere with acid-base parameters such as albumin levels. Therefore, in order to assess the independent association of acid-base variables and cytokine levels, we performed a principal component analysis (PCA) using the simplified acute physiology score (SAPS)3 score as a way of quantifying illness severity and including acid-base components that had an important impact on cytokine levels in the initial GLM analysis. We included SIG, SID_a_, lactate, albumin and SAPS3 score in the PCA. Phosphate and PCO2 were not included in the PCA due to weak associations using the GLM. Variables were log-transformed before creation of principal components due to expected differences in variances. The number of components was selected in order to keep over 70% of the variation and through inspection of a scree plot. Components were then rotated using the varimax method. The resulting scores of the principal component analysis were then used for linear regression with selected cytokine levels (IL6, IL10, IL8 and MCP1).

### Organ dysfunction analysis

We evaluated the association between cytokine measurements at admission and occurrence of AKI and shock in the first 48 hours after ICU admission through logistic regression (see Additional file 1 for details). A *P*-value <0.05 was considered significant for all analyses. All analyses were performed using R Project version 2.15.2 using *pROC*, *car, MASS, Rcmdr, binonTools, PredictABEL, earth, psych, GPArotation* and *ggplot2* packages.

## Results

Clinical and laboratory features for the cohort are shown in Table 1. In total 44 patients were admitted due to sepsis. The most common sources of infection were pneumonia (13 patients), bloodstream (11 patients, 5 of whom had infection related to an intravascular device), urinary tract (4 patients) and intra-abdominal (3 patients). Surgery for source control was required in two patients with abdominal infection. All intravascular devices associated with bloodstream infection were removed. Reasons for admission in non-septic patients included acute pulmonary edema (10 patients), stroke (6 patients), pulmonary embolism (3 patients), subarachnoid hemorrhage (3 patients), postoperative admission due to major surgery (3 patients) and other reasons (18 patients). No patients received albumin prior to ICU admission. A total of 20 patients died in the ICU and 26 during the hospital stay. Additional data on the population are given in Additional file 1.

**Table 1:** Characteristics of all 87 included patients

**Demographic features**	**Value**
Age, years	52 (35 to 60)
Male, number of patients (%)	46 (53%)
Simplified acute physiology score 3	52 (41 to 67)
Sequential organ failure assessment score at admission	5 (2 to 9)
**Laboratory data at admission**	
Hemoglobin, g/dL	11 (2.32)
Hematocrit (%)	33.4 (6.8)
Leucocytes, cells/mm^3^	11,070 (7,400 to 16,000)
Platelets, units/mm^3^	174 (130 to 260)
Na, mEq/L	140 (9.2)
K, mEq/L	4.1 (0.9)
Cl, mEq/L	106 (9.4)
P, mg/dL	2.24 (0.99)
Creatinine, mg/dL	1.24 (0.8 to 2.8)
pH	7.39 (7.33 to 7.41)
pCO_2_, mmHg	38.72 (10.8)
HCO_3_ ^-^, mEq/L	21.83 (5.0)
SBE, mEq/L	-2.9 (5.5)
SIG, mEq/L	8.92 (5.13)
SIDa, mEq/L	40.7 (4.89)
Lactate, mEq/L	1.55 (1.11 to 2.22)
Albumin, g/dL	2.91 (0.73)
**Specific reasons for admission, number of patients (%)**	
Sepsis	27 (31%)
Surgery	3 (3%)
**Organ dysfunction and need for support, number of patients (%)**	
Shock	25 (28%)
Norepinephrine dose <0.3 mcg/kg/min	8 (9%)
Norepinephrine dose ≥0.3 mcg/kg/min	18 (20%)
Steroid use for shock	18 (20%)
Use of mechanical ventilation	19 (22%)
PO2/FiO2 ratio <100	1 (1%)
Acute kidney injury	27 (31%)
Need for renal replacement therapy	25 (29%)
Continuous method	14 (16%)
**Outcomes, number of patients (%)**	
ICU mortality	20 (23%)
Hospital mortality	26 (30%)
**Cytokine levels, pg/mL**	
IL1β	2.1 (1.2 to 4.1)
IL1RA	25.4 (12 to 59.7)
IL2	7.5 (4.9 to 12.8)
IL4	7.18 (2.41 to 18.76)
IL6	45.76 (11.2 to 110)
IL7	13.3 (7.0 to 20.9)
IL8	36.3 (19.3 to 81.8)
IL9	2.0 (0.2 to 3.7)
IL10	17.7 (7.4 to 57.9)
IL17	3.5 (1.8 to 8.1)
TNFα	25.7 (12.5 to 45.0)
IFNα	66.3 (52.5 to 93.2)
IFNγ	9.4 (5.4 to 16.8)
VEGF	161 (104 to 311)
CXCL1	1275 (901 to 2,338)
MCP1	401 (226.5 to 1,450.0)

Data are shown as mean (SD) or median (IQ) unless stated otherwise. PO2/FiO2, partial pressure of dissolved oxygen/inspired oxygen fraction; VEGF, vascular endothelial growth factor; CXCL, chemokine CXC motif ligand; MCP, monocyte chemoattractant protein.

Results for the GLM for each mediator and acid-base variable are shown in Table 2. SID_a_ was negatively associated with plasma IL10 and IL17 whereas SIG was positively associated with IL6, IL8, IL10 and TNFα. Lactate was positively associated with IL8 and IL17. Phosphate was negatively associated with IL6. Higher albumin values were associated with lower values of IL6, IL7, IL8, IL10, TNFα and IFNα (Table 1). There was no association between PCO2 and any measured cytokine on GLM analysis.

**Table 2:** Results of the GLM of each cytokine and components of acid-base status (only significant values are shown)

**Cytokine**	**SID**_**a**_		**SIG**		**Lactate**		**Albumin**		**Phosphate**	
	**Est**	***P***	**Est**	***P***	**Est**	***P***	**Est**	***P***	**Est**	***P***
**IL6**	NS	NS	0.033	0.004	NS	NS	-0.23	0.004	-0.12	0.022
**IL7**	NS	NS	NS	NS	NS	NS	-0.13	0.035	NS	NS
**IL8**	NS	NS	0.016	0.049	0.02	0.036	-0.177	0.002	NS	NS
**IL10**	-0.031	0.006	0.03	0.005	NS	NS	-0.22	0.005	NS	NS
**IL17**	-0.045	0.015	NS	NS	0.052	0.035	NS	NS	NS	NS
**TNFα**	NS	NS	0.01	0.030	NS	NS	-0.119	0.037	NS	NS
**IFNα**	NS	NS	NS	NS	NS	NS	-0.070	0.045	NS	NS

There was no association between pCO2 and any cytokine levels. Estimates (Est) are for logarithmic relation. NS = Non significant.

**Figure 1:**
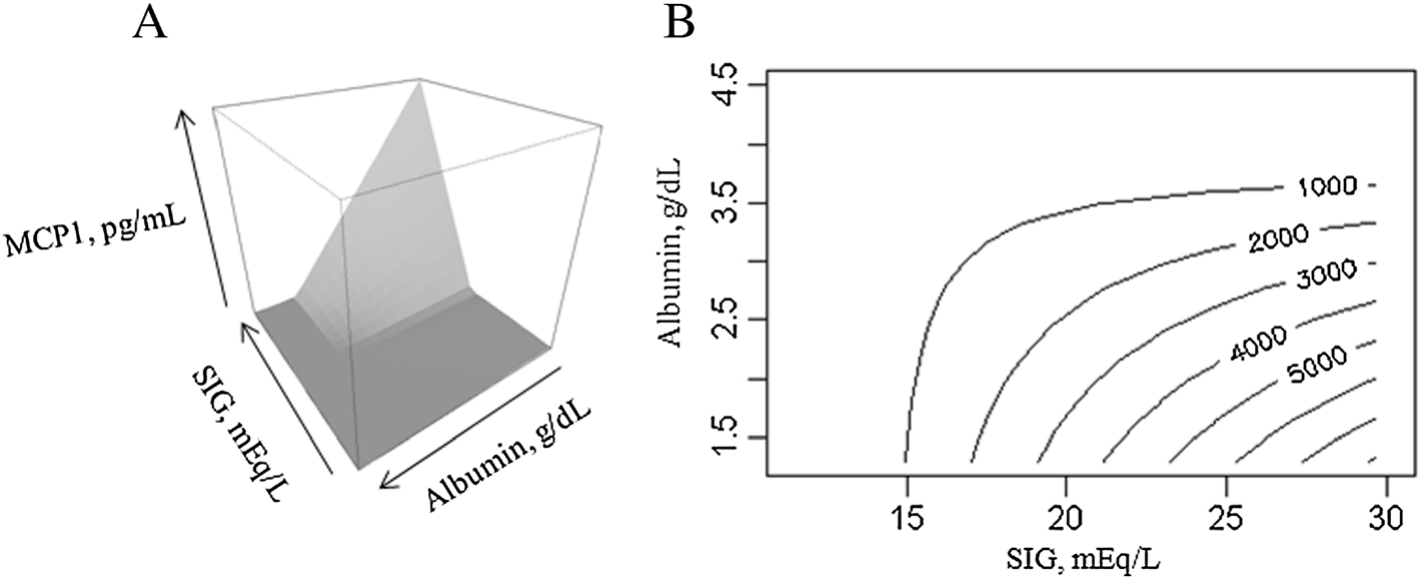
**Associations between albumin, strong ion gap (SIG) and monocyte chemoattractant protein (MCP)1. (A)** Perspective plot for the association between albumin, SIG and MCP1 levels. **(B)** Contour plot for the same association with MCP1 levels isopleths. Note the addictive effect of low albumin and high SIG values on MCP1 levels.

**Figure 2:**
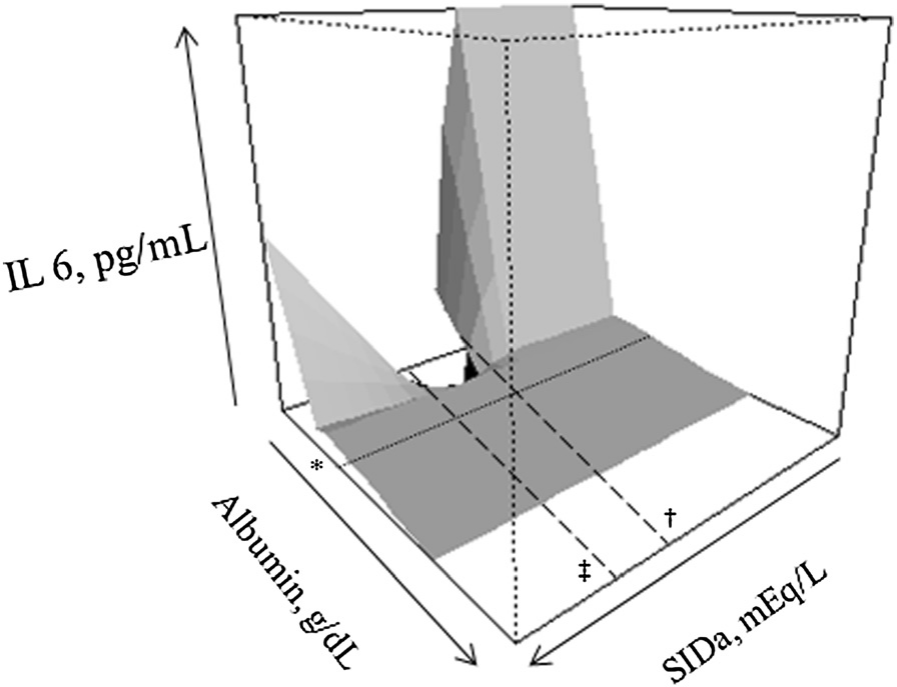
**Perspective plot for the association between albumin, apparent strong ion difference (SID**_**a**_**) and IL6 levels.** Note that for the most frequent SID_a_ range (marked by dashed lines from 36 (†) to 45 (‡) mEq/L) there was no relationship with IL6 levels and SID_a_. For abnormal values (both high and low) IL6 tended to increase. This association did not occur for higher albumin levels (*above 2.2 g/dL).

Results for the MARS analysis are shown in detail in Additional file 1. MARS models were built for IL6, IL8, IL10 and MCP1. We observed a negative association between albumin and all these mediators. The association between mediators and components of acid base (for example, SIG) occurred mostly when serum albumin concentrations were low (see figures in Additional file 1). Two examples are shown in Figure 1 and Figure 2. Figure 1 shows that higher SIG values were only associated with increased MCP1 when albumin concentrations were low (see Figure 1 legend for details). Figure 2 shows that changes in IL6 concentrations due to SID_a_ variation did not occur at higher albumin concentrations.

**Figure 3:**
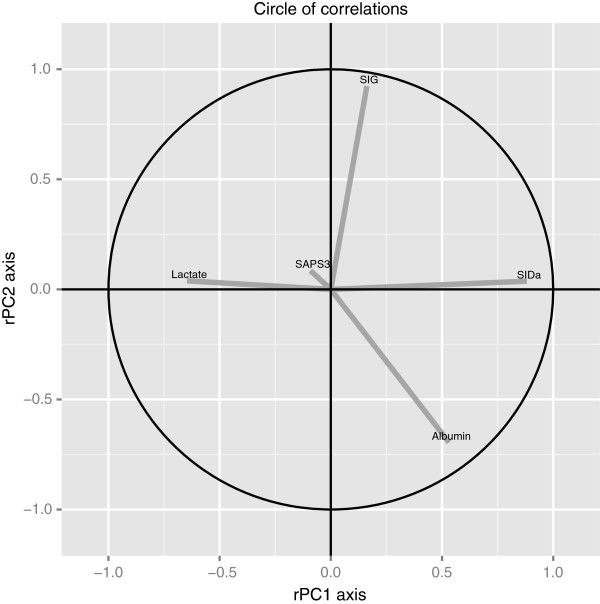
**Circle of correlations showing the relative impact of simplified acute physiology score 3 (SAPS3), strong ion gap SIG, strong apparent ion difference (SID**_**a**_**), albumin and lactate on principal components 1 and 2 (rPC1 and rPC2), which are displayed on the x and y axis, respectively.** The projection of the vector representing each variable is proportional to the load on the principal score.

Results of PCA analysis are shown in Table 3. Three principal components were selected for analysis (PC 1 to 3), accounting for 78% of all variance. Loadings for each component of acid-base status in each PC score are shown in Table 3. PC1 was largely positively determined by SID_a_ and albumin, and negatively determined by lactate. PC2 was positively determined by SIG and negatively determined by albumin. Both PC1 and PC2 were relatively independent of SAPS3 score, whereas PC3 mostly reflected SAPS3 score. Figure 3 shows the correlation circle of the importance of SAPS3, SID_a_, SIG, lactate and albumin on PC1 and PC2. Association between principal components and levels of IL6, IL8, IL10 and MCP1 are shown in Table 4; increased values of PC1 were associated with decreased inflammation, whereas increased values of PC2 and PC3 were associated with increased inflammation.

**Table 3:** Results of the principal component analysis (only principal components used in analysis are shown)

	**PC1**	**PC2**	**PC3**
Eigenvalue	1.51	1.35	1.02
Proportion of variance	0.30	0.27	0.20
Cumulative variance	0.30	0.57	0.78
**Loadings**			
Simplified acute physiology score 3 (SAPS3)	-0.09	0.09	0.98
Albumin	0.53	-0.70	-0.12
Strong apparent ion difference (SIDa)	0.88	0.04	0.07
Strong ion gap (SIG)	0.16	0.92	0.05
Lactate	-0.65	0.04	0.19

Note that principal component (PC)1 was largely positively determined by SIDa and albumin, and negatively determined by lactate. PC2 is positively determined by SIG and negatively determined by albumin. Both PC1 and PC2 are relatively independent of SAPS3 score. PC3 reflects mostly SAPS 3 score. PC = principal component.

**Table 4:** Results of the linear regression between principal component (PC) scores and selected cytokine levels

	**PC1**			**PC2**			**PC3**		
	**Est**	***P***	***r***^**2**^	**Est**	***P***	***r***^**2**^	**Est**	***P***	***r***^**2**^
**IL6**	-0.30	0.001	0.10	0.32	<0.001	0.12	0.34	<0.001	0.14
**IL8**	-0.21	<0.001	0.12	0.18	0.002	0.09	0.24	<0.001	0.17
**IL10**	-0.27	<0.001	0.14	0.23	0.001	0.10	0.24	<0.001	0.11
**MCP1**	-0.14	0.016	0.06	NS	NS	NS	0.16	0.003	0.08

Cytokine values were logarithmized for regression. Est, estimate; MCP1, monocyte chemoattract protein 1. NS = Non significant.

In logistic regression analyses the only association with AKI that remained significant was for the chemokine MCP1 (*P* = 0.039). Similarly, shock was associated with IL8 (*P* = 0.002); whereas other mediators did not remain significant in the model. See Additional file 1 for details.

## Discussion

In this study of critically ill patients we analyzed the relationship between acid-base variables, inflammatory mediators and end-organ failures (AKI and shock) using three different statistical methods. We observed important associations between acid-base status and inflammation that to our knowledge have not be described previously. Our main objective was to analyze the association between components of acid-base status and plasma cytokine concentrations. As shown in Table 2, there was an inverse association between albumin and concentrations of several cytokines. There are several possible explanations for this finding. First, albumin is known to bind cytokines (as well as other proteins), thereby reducing plasma concentrations [16]. Second, albumin is a negative acute phase protein; it is therefore expected that patients with more severe disease and therefore more intense inflammatory response will present with lower serum albumin concentrations and higher plasma cytokine concentrations. However, our PC analysis indicates that the relationship between albumin and cytokines is independent of disease severity. A third hypothesis is that albumin exerts a direct effect on cytokine production [17]. Bar-Or has suggested that albumin could decrease production of TNF and IFNγ in peripheral blood monocytes [17]. In agreement, we also found an inverse relation between albumin and TNF concentrations. Albumin infusion has also been found to reduce the inflammatory response and improve microcirculation in a model of hemorrhagic shock [22].

As the major extracellular weak acid, albumin is a crucial component of acid-base physiology especially in the critically ill. Changes in serum albumin concentrations are known to impact acid-base balance [19]. There may be an important interplay between albumin and SIG. Gómez *et al.* reported that analbuminemic rats usually have decreased SID_a_ and increased SIG, speculating that anionic proteins are produced in the place of albumin and therefore, increasing SIG would be a physiologic response to hypoalbuminemia [23]. Interestingly, we were only able to find a clear association between SIG and inflammatory markers when serum albumin concentrations were low.

Similarly, in the MARS analysis we observed an almost universal association between higher albumin concentrations and lower mediator concentrations. Moreover, higher albumin appeared to reduce the association between changes in components of acid-base status and mediator levels. As seen in Figure 1, for example, higher SIG was only associated with higher MCP1 concentration only when albumin was low. Also, no association between SID_a_ variations and IL6 was noted when the albumin concentration was greater than 2.2 g/dL (Figure 2). Although our study was only observational and therefore cannot evaluate a causal or therapeutic role for albumin we believe that this finding is unlikely to be solely explained by the *negative-phase* role of albumin. Given the results of a recent meta-analysis showing that albumin (compared to crystalloid) for resuscitation appears to improve survival among septic patients [24], the potential immune-modulating effects of albumin should be examined. The results of the Albios trial of albumin supplementation for sepsis indicate that positive effects of albumin are limited to patients with shock [25]. Does albumin play a role in actively scavenging inflammatory mediators? Can this effect help explain its potential benefit in septic shock? These questions cannot be answered by our study but our results raise these intriguing possibilities.

A positive association between SIG and various plasma mediator concentrations was found. Higher SIG was associated with higher levels of IL6, IL8, IL10 and TNF. These results suggest that patients with increased unmeasured anions may be those with more intense immune activation and, therefore, higher circulating cytokines and chemokines. This finding may be useful for further studies in helping to select the target population for immunological therapies in the critically ill. SIG, however, was not associated with increased levels of MCP1 in our GLM analysis but was apparent in the alternative analysis using MARS. In the MARS models we noted a positive association between SIG and IL8, IL10 and MCP1 (Figure 2; see also Additional file 1: Figures S4 and S5) that appeared to be modulated by albumin.

Our PCA analysis suggests that despite the fact that illness severity is associated with increased inflammation (as seen on the association between PC3 values and cytokine levels), there is a strong association between acid-base components and inflammation that is independent of a global assessment of the SAPS3 score. PCA 1 was largely determined by SID_a_, albumin and lactate, with a positive effect for SID_a_ and albumin and a negative effect for lactate. Higher PC1 scores were associated with decreased inflammation, allowing one to conclude that higher SID_a_, higher albumin and lower lactate may be associated with lower cytokine levels. Using the same rationale, SIG had a high positive effect and albumin had a negative effect on PC2, which was positively associated with increased cytokine levels. Therefore, it is conceivable that high SIG and low albumin are associated with increased inflammation. The presence of a large effect for albumin in both PC1 and PC2 highlights its potential role in modulating the association between abnormal SID_a_ and SIG values and inflammation.

Finally, we used logistic regression to evaluate the independent role of specific cytokines on relevant clinical scenarios, namely the development of AKI and shock. Due to the limited number of events, we restricted variable inclusion in the model [26]. Only plasma MCP1 was positively associated with AKI and only IL8 was positively associated with shock. This is in agreement with a previous study of septic patients from Bozza *et al.*, in which IL8 and MCP1 had good correlation with organ dysfunction as evaluated by global SOFA scores (*R* 0.50 and 0.43, respectively) [20].

There are several limitations in our analysis. Most important, it was performed on a small sample of critically ill patients. Second, we had only one sample for each patient, therefore, serial sample analysis could not be performed. Third, the cytokine levels, especially IL6, were low when compared to previous reports [27], although recent studies have shown values for IL6 similar to ours [28]. Finally, we have no data regarding the amount or type of crystalloid used during resuscitation, although Lactated Ringer’s is the fluid of choice at our institution. Strengths of our analysis include the use of robust statistical analysis that takes into account the complex interplay between variables.

## Conclusion

Acid-base variables (especially increased SIG and decreased albumin) on admission to ICU are associated with immunological activation. Albumin may modulate the circulating concentrations of various cytokines in a complex interplay with other components of acid-base status. The association between acid-base components and inflammation cannot be solely explained based on illness severity.

## Key messages

• There is a strong interplay between acid-base variables and immunological activation.

• Albumin may modulate circulating concentrations of several cytokines in a complex interplay with other components of acid-base status, such as SIG or SID_a_.

• Higher SIG values appear to be associated with increasing inflammation.

• The association between acid-base components and inflammation cannot be explained only by illness severity.

## Abbreviations

AKI: acute kidney injury; CXCL: chemokine CXC motif ligand; GLM: generalized linear model; IFN: interferon; IL: interleukin; MARS: multivariate adaptive regression with splines; MCP: monocyte chemoattractant protein; PCA: principal component analysis; SAPS 3: simplified acute physiology score 3; SID_a_: apparent strong ion difference; SID_e_: effective strong ion difference; SIG: strong ion gap; SOFA: sequential organ failure assessment score; TNF: tumor necrosis factor; VEGF: vascular endothelial growth factor.

## Competing interests

The authors declare that they have no competing interests.

## Authors’ contributions

FGZ: conception and design, data collection and statistical analysis, manuscript draft and final approval of the manuscript. JAK: study design, statistical analysis, manuscript draft and approval of the final version of the manuscript. MP: study design, data collection, statistical analysis, manuscript review and final approval of the manuscript draft. OTR: study design, statistical analysis, manuscript review and final approval of the manuscript draft. HVB: acquisition of data and processing of samples, revision of the manuscript, final approval of the manuscript. HPS: study design, acquisition of data and processing of samples, revision of the manuscript, final approval of the manuscript. LMDCN: acquisition of data and processing of samples, revision of the manuscript, final approval of the manuscript. FPS: study design, collecting and processing of samples, revision of the manuscript, final approval of the manuscript. All authors approved the final version of the manuscript.

## Supplementary Material

Additional file 1 Additional patient information, results for organ dysfunction regression and results for multi-adaptive regression splines. Click here for file
