# Supplementary material for: Relationship between acid–base status and inflammation in the critically ill
Source: Crit Care. 2014 Jul 17;18(4):R154. doi: 10.1186/cc13993 (PMC4223545; doi:10.1186/cc13993)
Supplement: Additional file 1 — Additional patient information, results for organ dysfunction regression and results for multi-adaptive regression splines. [file cc13993-S1.docx]

**Relationship between acid-base status and inflammation in the critically ill**

***Additional File***

**1. Logistic regression for organ dysfunction results:**

**a. Definition of organ dysfunction:**

Shock was defined as need for vasopressors at any dose for at least 30 minutes during the first 48 hours after admission. Patients admitted using vasopressors and in whom the drug could not be withheld in 30 minutes after admission were also considered to have shock.

AKI was defined as any increase in serum creatinine of at least 0.3 mg/dL within 48 hours or an absolute increase of 50% over baseline creatinine or need for renal replacement therapy [1].

**b. Caracteristics of patients with shock and AKI:**

25 patients fulfilled the shock definition and 27 met the definition of AKI. The sTable 1 and sTable 2 shows the characteristics of both groups and cytokine levels. All cytokine levels are in pg/mL.

**b1. Patients with shock versus without shock (sTable 1)**

|  | Shock  (n = 25) | No Shock  (n = 62) | P |
| --- | --- | --- | --- |
| Age, years | 46 [35-60] | 52 [37.25-60] | 0.42 |
| Male (%) | 12 (48%) | 38 (61%) | 0.73 |
| SAPS3 | 70 [58-83] | 47.5 [38.5-57] | < 0.001 |
| SOFA score at admission | 10 [8-12] | 3 [2-5] | < 0.001 |
| Admission Laboratorial Data | | |  |
| Hemoglobin, g/dL | 10.3 (2.0) | 11.35 (2.3) | 0.057 |
| Hematocrit (%) | 31 (5.7) | 34 (7.0) | 0.218 |
| Leucocytes, cells/mm^3^ | 12150 [6050-16720] | 10880 [7420-15220] | 0.966 |
| Platelets, units/mm^3^ | 162 [108-276] | 184 [142-251] | 0.475 |
| Na, mEq/L | 140.1 (9.1) | 141 (9.3) | 0.658 |
| K, mEq/L | 4.02 (1.1) | 4.17 (0.83) | 0.520 |
| Cl, mEq/L | 106 (7.9) | 106 (10.0) | 0.980 |
| P, mg/dL | 2.3 (1.4) | 2.2 (0.7) | 0.605 |
| Creatinine, mg/dL | 1.34 [0.83-2.87] | 1.14 [0.80-2.35] | 0.985 |
| pH | 7.34 [7.22-7.39] | 7.39 [7.36-7.41] | 0.004 |
| pCO_2_, mmHg | 41.3 [34.4-45.8] | 36.4 [30.7-41.9] | 0.092 |
| HCO_3_^-^, mEq/L | 20.5 (4.1) | 22.3 (5.3) | 0.099 |
| SBE, mEq/L | -4.3 (5.9) | -2.2 (5.1) | 0.107 |
| SIG, mEq/L | 8.9 (4.2) | 8.9 (5.4) | 0.963 |
| SIDa, mEq/L | 38.7 (4.44) | 41.5 (4.88) | 0.015 |
| Lactate, mEq/L | 1.89 [1.44-2.78] | 1.38 [1.00-1.89] | 0.010 |
| Albumin, g/dL | 2.52 (0.63) | 3.07 (0.71) | <0.001 |
|  | | |  |
| Admission due to sepsis | 23 (92%) | 2 (3%) | <0.001 |
| AKI | 13 (48%) | 12 (20%) | 0.015 |
| Outcomes | | |  |
| ICU mortality | 10 (40%) | 10 (16%) | <0.001 |
| Hospital mortality | 14 (56%) | 12 (20%) | 0.001 |
| Cytokine concentration | | |  |
| IL1β | 3.2 [1.9-5.6] | 1.8 [1.2-3.5] | 0.023 |
| IL1RA | 51.0 [17.8-130.0] | 21.6 [7.4-46.1] | 0.001 |
| IL2 | 10.0 [7.2-14.8] | 7.2 [4.6-10.3] | 0.032 |
| IL4 | 15.5 [2.4-29.5] | 4.2 [2.4-14.8] | 0.056 |
| IL6 | 133.0 [21.8-1076] | 32.6 [9.2-63.4] | <0.001 |
| IL7 | 20.0 [14.0-34.5] | 11.0 [5.3-15.9] | <0.001 |
| IL8 | 82.5 [41.7-297] | 30.2 [11.8-54.1] | <0.001 |
| IL9 | 3.3 [1.4-4.3] | 1.74 [0.1-3.3] | 0.061 |
| IL10 | 55.8 [25.3-101.0] | 11.9 [4.3-26.1] | <0.001 |
| IL17 | 7.7 [3.95-10.5] | 2.6 [1.5-5.2] | < 0.001 |
| TNFα | 44.8 [23.1-95.4] | 19.7 [10.3-33.1] | <0.001 |
| IFNα | 92. [62.2-104.0] | 64.3 [43.2-82.6] | 0.004 |
| IFNγ | 16.7 [9.8-36.2] | 7.9 [4.7-14.8] | 0.001 |
| VEGF | 236 [173-386] | 135 [87.9-286.2] | 0.004 |
| CXCL1 | 1213 [930-2644] | 1277 [898-2208] | 0.596 |
| MCP1 | 1267 [517.0-2339] | 325.5 [196.0-560.2] | 0.002 |

|  | AKI Group  (n = 27) | No-AKI Group  (n = 60) | P |
| --- | --- | --- | --- |
| Age, years | 53 [35-66] | 51.5 [37.35-58.50] | 0.755 |
| Male (%) | 17 (62%) | 29 (48%) | 0.30 |
| SAPS3 | 63 [52.5-79] | 48.5 [38-58] | < 0.001 |
| SOFA score at admission | 9 [4.5-12] | 3 [2-7.25] | < 0.001 |
| Admission Laboratorial Data | | |  |
| Hemoglobin, g/dL | 10.7 (2.36) | 11.2 (2.3) | 0.330 |
| Hematocrit (%) | 32 (6.55) | 34 (6.94) | 0.218 |
| Leucocytes, cells/mm^3^ | 10520 [7020-16380] | 11550 [7500-14410] | 0.901 |
| Platelets, units/mm^3^ | 162 [116-257] | 187 [138-255] | 0.34 |
| Na, mEq/L | 140.7 (9.1) | 140.8 (9.39) | 0.99 |
| K, mEq/L | 4.44 (1.04) | 3.99 (0.82) | 0.057 |
| Cl, mEq/L | 106.1 (9.6) | 106 (9.4) | 0.957 |
| P, mg/dL | 4.5 (2.36) | 3.5 (1.32) | 0.058 |
| Creatinine, mg/dL | 1.98 [1.06-3.16] | 1.10 [0.73-2.09] | 0.03 |
| pH | 7.35 [7.28-7.40] | 7.39 [7.35-7.42] | 0.010 |
| pCO_2_, mmHg | 36.9 [32.5-44.9] | 36.8 [31.9-42.1] | 0.40 |
| HCO_3_^-^, mEq/L | 20.2 (4.6) | 22.5 (5.1) | 0.043 |
| SBE, mEq/L | -5.75 (5.5) | -1.67 (4.9) | 0.002 |
| SIG, mEq/L | 10.4 (5.2) | 8.2 (4.9) | 0.080 |
| SIDa, mEq/L | 40.0 (4.50) | 41.0 (5.07) | 0.398 |
| Lactate, mEq/L | 1.67 [1.33-3.78] | 1.44 [1-2] | 0.08 |
| Albumin, g/dL | 2.6 (0.74) | 3.0 (0.72) | 0.028 |
|  |  |  |  |
| Admission due to sepsis | 18 (66%) | 25 (41%) | 0.054 |
| Shock | 13 (48%) | 12 (20%) | 0.015 |
| Outcomes | | |  |
| ICU mortality | 15 (55%) | 5 (8%) | <0.001 |
| Hospital mortality | 18 (66%) | 8 (13%) | <0.001 |
| Cytokine concentration | | |  |
| IL1β | 2.0 [0.6-3.6] | 2.1 [1.3-5.1] | 0.342 |
| IL1RA | 22.0 [12.0-57.6] | 33.11 [12.3-61.6] | 0.873 |
| IL2 | 7.5 [4.7-10.7] | 7.7 [5.1-13.6] | 0.881 |
| IL4 | 8.7 [2.4-16.0] | 6.6 [2.4-20.7] | 0.930 |
| IL6 | 83.6 [35.2-415.0] | 29.6 [9.5-68.3] | 0.004 |
| IL7 | 14.7 [11.3-27.6] | 12.3 [6.5-18.7] | 0.070 |
| IL8 | 55.3 [35.9-202] | 31.0 [16.3-64.4] | 0.004 |
| IL9 | 2.3 [0.1-3.5] | 1.8 [0.2-4.0] | 0.941 |
| IL10 | 53.7 [17.5-80.5] | 12.6 [4.8-205] | 0.003 |
| IL17 | 3.6 [2.0-8.1] | 3.4 [1.8-7.3] | 0.768 |
| TNFα | 28.5 [19.7-47.8] | 21.8 [11.3-36.2] | 0.108 |
| IFNα | 62.2 [54.1-88.2] | 66.6 [46.8-93.2] | 0.908 |
| IFNγ | 10.1 [6.0-18.2] | 8.8 [5.4-16.5] | 0.653 |
| VEGF | 173 [136.5-357.5] | 142.5 [90.6-287.2] | 0.190 |
| CXCL1 | 1213 [803-2362] | 1277 [959-2252] | 0.620 |
| MCP1 | 947 [277-2502] | 354 [220-717.2] | 0.030 |

**b1. Patients with AKI versus without AKI (sTable 2)**

**c. Statistical analysis:**

Two models were built using logistic regression: one to evaluate mediators associated with AKI and other for presence of shock. All mediators that were associated with the outcome of interest in univariate analysis (using a cutoff of p < 0.25 [2]) were included in the regression. Mediators were entered as variables and the occurrence of organ dysfunction as binary outcomes [3]. A stepwise backwards approach using Bayesian information criterion (BIC) was then employed in order to obtain the final model. Collinearity was assessed by measurement of variance inflation factor (VIF). A VIF greater than 2.5 was considered suggestive of collinearity. Calibration of AKI and shock models were assessed using the Hosmer-Lemeshow test [4].

**d. Results**

Twenty five patients met our definition of shock (required vasopressors for more than 30 minutes in any period of the first 48 hours after admission). Most of the patients with shock were already receiving vasopressors at ICU admission (15 patients). Comparisons between patients with and without shock are shown in sTable 1, above. Patients with shock were sicker, as attested by greater SAPS3 and SOFA scores. Patients with shock had lower pH (7.34 [7.22-7.39] versus 7.39 [7.36-7.41]; p = 0.004), lower SIDa (38.7 ±4.44 versus 41.5 ±4.88 mEq/L; p = 0.015) and higher lactate (1.89 [1.44-2.78] versus 1.38 [1.00-1.89] mEQ/L; p = 0.01). Albumin was lower in patients with shock (2.52 ± 0.63 versus 3.07 ± 0.71 g/dL; p < 0.001). As expected, patients with shock had higher ICU and hospital mortality (sTable 1). The large majority of patients with shock were admitted due to sepsis (92%, see sTable 4 bellow). All mediators with the exception of CXCL1, IL4 and IL9 were increased in patients with shock compared to patients without shock (sTable 1).

Patients developing AKI had more severe disease at admission as assessed by SAPS3 and SOFA scores. Those developing AKI likewise had higher serum concentrations of creatinine on admission (1.98 [1.06-3.16] versus 1.10 [0.73-2.09] mg/dL; p = 0.03), lower arterial blood pH (7.35 [7.28-7.40] versus 7.39 [7.35-7.42]; p = 0.01), lower plasma bicarbonate (20.2 ± 4.6 versus 22.5 ± 5.1 mEq/L; p = 0.04), lower arterial blood base excess (-5.75 ± 5.5 versus -1.67 ± 4.9 mEq/L; p = 0.02) and lower serum albumin (2.6 ± 0.74 versus 3.0 ± 0.72 g/dL; p = 0.02) when compared to patients not developing AKI. There were more patients with shock among those with AKI compared those without (48% versus 20%; p = 0.01). Both ICU and hospital mortalities were higher for patients with AKI (Table 2). In univariate analysis, patients developing AKI had higher plasma concentrations of IL6, IL8, IL10 and MCP1 (sTable 2).

In multivariate analyses the only association with AKI that remained significant was for the chemokine MCP1 (p = 0.039). Albeit the calibrated (Hosmer-Lemeshow p = 0.16), AUC for the model was low 0.64 (95% CI 0.51-0.77), as well as coefficient of determination (0.08). Similarly, shock was associated with IL8 (p = 0.002); whereas other mediators did not remain significant in the model.

**2. Results for the MARS analysis:**

MARS analysis was performed on R using *earth package.* A MARS model was built for IL6, IL8, IL10 and MCP1. Dependent variables included on the MARS model were PCO_2_, SID_a_, SIG, albumin and phosphate levels. Code for each analysis on R for eartch package is provided bellow, followed by the results and a brief discussion:

>> model<-earth(CYTOKINE ~ PCO2 + SIDA + SIG + LACTMOL + ALBUM + PI, degree=5, data=db) # Main code for the analysis. CYTOKINE was replaced by the name of the variable for each cytokine included on the analysis.

>> plotmo(terra) # Used to obtain graphical results

>> summary(terra,digits=2,style="pmax") #Used to obtain the summary of the model

1. IL6 model: *5e+02 - 302 * pmax(0, ALBUM - 2) + 14238 * pmax(0, 2 - ALBUM) - 564 * pmax(0, 42 - PCO2) * pmax(0, 2.1 - ALBUM) - 3759 * pmax(0, SIDA - 36) * pmax(0, 2 - ALBUM) + 4357 * pmax(0, SIDA - 40) * pmax(0, 2 - ALBUM) - 66780 * pmax(0, SIDA - 45) * pmax(0, LACTMOL - 3.2) * pmax(0, ALBUM - 2) - 365 * pmax(0, 45 - SIDA) * pmax(0, LACTMOL - 3.2) * pmax(0, ALBUM - 2) + 290 * pmax(0, SIG - -2.2) * pmax(0, LACTMOL - 3.2) * pmax(0, ALBUM - 2) * pmax(0, PI - 0.23)*

Therefore, there was a negative association between albumin levels and IL6 for albumin values above 2 g/dL. Other two significant interactions were between IL6 levels and the relationship between PCO2 and albumin (sFigure1, bellow) and between SIDa and albumin (sFigure2, bellow – a modified version is shown on the manuscript). Higher albumin levels were protective against the association between high PCO2 or changes in SIDa and IL6 levels.


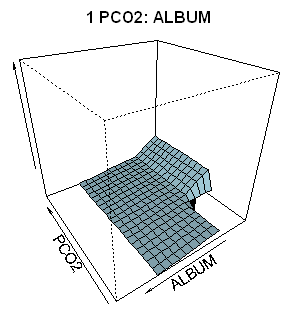

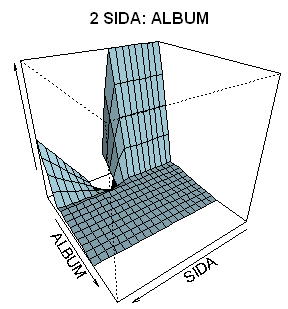


sFigure 1 sFigure 2

1. IL8 model: 1.5e+02 - 74 * pmax(0, ALBUM - 2) - 188 * pmax(0, SIDA - 36) * pmax(0, 2 - ALBUM) - 178 * pmax(0, 36 - SIDA) * pmax(0, 2 - ALBUM) + 899 * pmax(0, SIDA - 40) * pmax(0, 2 - ALBUM) - 649 * pmax(0, SIG - 12) * pmax(0, 2 - ALBUM) + 1004 * pmax(0, SIG - 12) * pmax(0, 2 - ALBUM) * pmax(0, PI - 2.3) + 6419 * pmax(0, SIG - 12) * pmax(0, 2 - ALBUM) * pmax(0, 2.3 - PI)

There was an association between higher albumin levels and IL8 levels. Higher albumin levels were also “protective” against a positive association between SID_a_ and IL8 and SIG and IL8 as shown in sFigure 3 and sFigure 4.


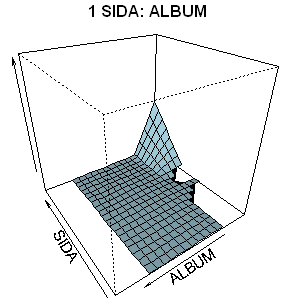

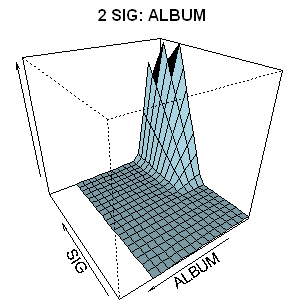


sFigure 3 sFigure 4

1. IL 10 model: -42 + 61 * pmax(0, 14 - SIG) + 2607 * pmax(0, ALBUM - 2.3) - 156 * pmax(0, SIG - -2.2) * pmax(0, ALBUM - 2.3) + 121 * pmax(0, SIG - 14) * pmax(0, ALBUM - 2) - 165 * pmax(0, 14 - SIG) * pmax(0, ALBUM - 2) + 4540 * pmax(0, SIG - 12) * pmax(0, 2 - ALBUM) * pmax(0, 2.3 - PI)

On this analysis, there was a U shaped associated between SIG and IL10, with both higher and lower SIG values associated independently with higher IL10 (the bending point was approximately 14 – sFigure 5). Higher albumin was also associated with lower IL10 levels but on a narrower range (between 2.0 and 2.5, with little effects for other albumin values). Again, the effect of SIG on IL10 levels was reduced by higher levels of albumin, as shown on sFigure 5.


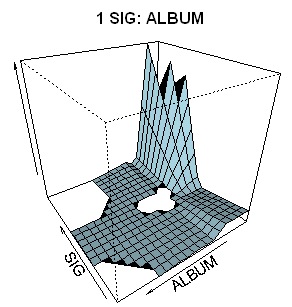


sFigure 5

1. MCP1 model: 5.8e+02 + 202 * pmax(0, SIG - 14) * pmax(0, 3.8 - ALBUM) + 154 * pmax(0, PCO2 - 30) * pmax(0, 41 - SIDA) * pmax(0, 3.8 - ALBUM) - 212 * pmax(0, PCO2 - 42) * pmax(0, 41- SIDA) * pmax(0, 3.8 - ALBUM) - 40 * pmax(0, PCO2 - 40) * pmax(0, 14 - SIG) * pmax(0, 3.8 - ALBUM) + 69 * pmax(0, PCO2 - 47) * pmax(0, 14 - SIG) * pmax(0, 3.8 - ALBUM) - 603 * pmax(0, 14 - SIG) * pmax(0, 3.8 - ALBUM) * pmax(0, 1.3 - PI) - 39 * pmax(0, PCO2 - 30) * pmax(0, 41 - SIDA) * pmax(0, SIG - 12) * pmax(0, 3.8 - ALBUM) - 4.3 * pmax(0, PCO2 - 30) * pmax(0, 41 - SIDA) * pmax(0, 12 - SIG) * pmax(0, 3.8 - ALBUM)

Although almost all components had an association with MCP1 levels, the strongest association was seen for the relationship between SIG and albumin (see Figure 2 of the main manuscript). Patients with high albumin levels tented to have low MCP1 values despite high SIG.

**3. Supplementary Table 3**: Comparison between septic and non-septic patients.

|  | Septic patients  (n=43) | Non-septic patients  (n=44) | P (between groups) |
| --- | --- | --- | --- |
| Age, years | 52 [33-64] | 51.5 [39.2-59.5] | 0.680 |
| Male (%) | 24 (56%) | 22 (50%) | 0.580 |
| SAPS3 | 60 [46-76] | 47.5 [37.25-53.75] | < 0.001 |
| SOFA score at admission | 8 [4-10] | 3 [1.25-5] | < 0.001 |
| Admission Laboratorial Data | | |  |
| Hemoglobin, g/dL | 10.4 (2.3) | 11.7 (2.1) | < 0.001 |
| Hematocrit (%) | 31 (6.3) | 36 (6.4) | < 0.001 |
| Leucocytes, cells/mm^3^ | 12500 [6900-17400] | 10600 [7400-13190] | 0.248 |
| Platelets, units/mm^3^ | 162 [110-276] | 190 [149-260] | 0.102 |
| Na, mEq/L | 138 (7.6) | 143 (10.3) | 0.042 |
| K, mEq/L | 4.2 (1.0) | 4 (0.8) | 0.390 |
| Cl, mEq/L | 105 (6.6) | 107.5 (11.4) | 0.160 |
| P, mEq/L | 2.38 (1.2) | 2.1 (0.73) | 0.200 |
| Creatinine, mg/dL | 1.66 [0.88-2.89] | 1.06 [0.75-2] | 0.149 |
| pH | 7.37 [7.28-7.4] | 7.4 [7.35-7.42] | 0.019 |
| pCO_2_, mmHg | 38 (12) | 39 (9) | 0.780 |
| HCO_3_^-^, mEq/L | 19.8 (4.3) | 24 (4.9) | < 0.001 |
| SBE, mEq/L | -5.1 (5.6) | -0.8 (4.4) | < 0.001 |
| SIG, mEq/L | 10 (5.0) | 7.9 (5.0) | 0.040 |
| SIDa, mEq/L | 39.3 (5.23) | 42.0 (4.17) | 0.008 |
| Lactate, mEq/L | 1.8 [1.3-3.2] | 1.3 [0.8-1.9] | 0.001 |
| Albumin, g/dL | 2.6 (0.62) | 3.2 (0.7) | < 0.001 |
| Organ Dysfunction | | |  |
| AKI | 18 (41%) | 9 (20%) | 0.054 |
| Shock | 23 (53%) | 2 (4%) | < 0.001 |
| Outcomes | | |  |
| ICU mortality | 16 (37%) | 4 (9%) | 0.02 |
| Hospital mortality | 19 (44%) | 7 (16%) | < 0.001 |
| Cytokines* | | |  |
| IL1β | 3.2 [1.4-5.5] | 1.6 [1.2-3.0] | 0.041 |
| IL1RA | 40.2 [18.3-66.2] | 13.8 [6.35-58.63] | 0.003 |
| IL2 | 8.46 [5.9-14.2] | 7.2 [4.3-10.3] | 0.10 |
| IL4 | 12.98 [2.41-29.53] | 2.41 [2.41-12.36] | 0.002 |
| IL6 | 83.66 [35.64-467] | 14.03 [5.65-49.89] | <0.001 |
| IL7 | 18.9 [12.5-28.6] | 9.7 [4.4-14.6] | <0.001 |
| IL8 | 70.7 [32.3-133.5] | 20.6 [9.7-39.5] | <0.001 |
| IL9 | 2.6 [0.2-4.6] | 1.7 [0.2-3.1] | 0.096 |
| IL10 | 53.59 [18.82-101] | 8.98 [3.94-16.17] | <0.001 |
| IL17 | 6.9 [2.6-10.3] | 2.41 [1.4-3.9] | <0.001 |
| TNFα | 35.3 [25.5-72.0] | 14.8 [9.0-25.9] | <0.001 |
| IFNα | 75.4 [58-97.8] | 62.4 [42.1-77.4] | 0.029 |
| IFNγ | 16.5 [8.9-32.7] | 6.8 [4.6-10.8] | 0.011 |
| VEGF | 236 [132.5-406] | 125 [74.6-179.5] | 0.001 |
| CXCL1 | 1143 [939-2186] | 1339 [875-2373] | 0.708 |
| MCP1 | 878 [322.5-2274.0] | 295.5 [168.8-517.5] | <0.001 |

**References:**

1. Kellum JA, Lameire N, Group ftKAGW: **Diagnosis, evaluation, and management of acute kidney injury: a KDIGO summary (Part 1)**. *Crit Care* 2013, **17**(1):204.

2. Hosmer Dw LS: **Applied Logistic Regression**, 2nd Edition edn: Wiley; 2000.

3. Zeger SL, Liang KY: **Longitudinal data analysis for discrete and continuous outcomes**. *Biometrics* 1986, **42**(1):121-130.

4. Lemeshow S, Hosmer DW: **A review of goodness of fit statistics for use in the development of logistic regression models**. *Am J Epidemiol* 1982, **115**(1):92-106.
